# Exogenous spermidine improves seed germination of sweet corn via involvement in phytohormone interactions, H_2_O_2_ and relevant gene expression

**DOI:** 10.1186/s12870-016-0951-9

**Published:** 2017-01-03

**Authors:** Yutao Huang, Cheng Lin, Fei He, Zhan Li, Yajing Guan, Qijuan Hu, Jin Hu

**Affiliations:** Seed Science Center, Institute of Crop Science, College of Agriculture and Biotechnology, Zhejiang University, 866 Yuhangtang Road, Hangzhou, 310058 China

**Keywords:** Gene expression, H_2_O_2_, Phytohormone, Seed germination, Spermidine, Sweet corn

## Abstract

**Background:**

The low seed vigor and poor field emergence are main factors that restricting the extension of sweet corn in China. Spermidine (Spd) plays an important role in plant growth and development, but little is known about the effect of Spd on sweet corn seed germination. Therefore the effect of exogenous Spd on seed germination and physiological and biochemical changes during seed imbibition of Xiantian No.5 were investigated in this study.

**Results:**

Spd soaking treatment not only improved seed germination percentage but also significantly enhanced seed vigor which was indicated by higher germination index, vigor index, shoot heights and dry weights of shoot and root compared with the control; while exogenous CHA, the biosynthesis inhibitor of Spd, significantly inhibited seed germination and declined seed vigor. Spd application significantly increased endogenous Spd, gibberellins and ethylene contents and simultaneously reduced ABA concentration in embryos during seed imbibition. In addition, the effects of exogenous Spd on H_2_O_2_ and MDA productions were also analyzed. Enhanced H_2_O_2_ concentration was observed in Spd-treated seed embryo, while no significant difference of MDA level in seed embryo was observed between Spd treatment and control. However, the lower H_2_O_2_ and significantly higher MDA contents than control were detected in CHA-treated seed embryos.

**Conclusions:**

The results suggested that Spd contributing to fast seed germination and high seed vigor of sweet corn might be closely related with the metabolism of hormones including gibberellins, ABA and ethylene, and with the increase of H_2_O_2_ in the radical produced partly from Spd oxidation. In addition, Spd might play an important role in cell membrane integrity maintaining.

**Electronic supplementary material:**

The online version of this article (doi:10.1186/s12870-016-0951-9) contains supplementary material, which is available to authorized users.

## Background

Sweet corn is extensively planted in China and many parts of the world due to its high sugar content and better flavor. China has become the world’s second largest sweet corn production country in 2004 [61]. However, its poor seed vigor easily causes unsatisfied field emergence, which severely limits the production of sweet corn [59]. The research on how to improve sweet corn by seed treatment is still lacking [35]. Therefore, how to improve the seed vigor of sweet corn is particularly important issue to be solved at present. Polyamines (PAs) including putrescine (Put), spermidine (Spd) and spermine (Spm) are small aliphatic polycationic nitrogenous compounds. They are considered as important modulators involving in plant growth and development regulation, such as flower, leaf and root differentiation, flower and fruit development, senescence, and germination of seed and pollen [25, 40, 42, 56]. Arginine decarboxylase (ADC), ornithine decarboxylase (ODC), S-adenosylmethionine decarboxylase (SAMDC), Spd synthase (SPDS) and Spm synthase (SPMS) are five key biosynthetic enzymes relating to polyamine biosynthesis in higher plants, and have already been studied for years in great detail [6]. Of which, SPDS catalyzes Spd synthesis; while cyclohexylamine (CHA), a polyamines biosynthesis inhibitor, inhibits directly the SPDS activity and leads to the decrease of Spd content. PAs are usually metabolized by diamine oxidase (DAO) and polyamine oxidase (PAO) [4]. Hydrogen peroxide (H_2_O_2_) is produced in the oxidation of PAs by DAO and PAO, which is considered to be necessary for plant-developmental processes [3, 43] .

In our previously study, endogenous Spd was found to be much more closely related with the seed quality including seed size, dry and fresh weight than Put or Spm during development of sweet corn [14]. Therefore, Spd was selected for seed soaking in this study. Spd is a major triamine among the three PAs and takes part in a variety of physiological processes. Exogenous Spd (0.01–1.0 mM) had been proven to spur alpha-amylase activity in maize seed and improve seed germination [20]. It was also used to enhance the rice seed vigor and reduce the toxicity of salt stress on the rice seed [58]. Tomato seed primed with Spd or Spm showed higher germination percentage, seedling vigor and anti-oxidative activity; while Put priming decreased anti-oxidative activities in tomato seeds [1]. Primed seed of white clover with Spd solution significantly enhanced the activities of alpha-amylases and beta-amylases, increased the contents of reducing sugar, fructose and glucose and improved transcript level of β-amylase gene. It suggested that the change of starch metabolism induced by Spd might be a possible reason for white clover seed vigor enhancement [36].

Gibberellins (GAs) and abscisic acid (ABA) are two important kinds of plant hormones in seed germination regulation. GAs mainly involves in seed dormancy breakdown. It had been proven to improve seed vigor and promote seed germination through enhancing endogenous hydrolase activities to weaken the barrier tissues such as the endosperm or seed coat, and through inducing mobilization of seed storage reserves and stimulating expansion of the embryo [9]. Hedden and Thomas [28] reported that terminal steps in GA biosynthesis were mainly catalyzed by gibberellin 20-oxidase (GA20ox) and gibberellin 3-oxidase (GA3ox); while the GA catabolism was catalyzed by gibberellin 2-oxidase (GA2ox). Low-temperature treatment up-regulated the GA3ox activity and down-regulated the GA2ox activity, inducing directly GAs accumulation and seed germination improvement of *Arabidopsis thaliana* [60]. The transcript level of GA biosynthetic gene *GA3ox2* increased 40-fold in after-ripened seeds (dormancy-broken) as compared with fresh seeds (dormant); whereas the GA-deactivating gene *GA2ox1* expressed at the highest levels in the highly dormant seeds of *Arabidopsis thaliana ecotype Cvi* (Cape Verde Islands) compared with non-dormant seeds [21].

On the contrary, abscisic acid (ABA) mainly takes part in seed dormancy maintenance. Dormancy is often released in ABA-deficient seeds; whereas over-expressions of genes relating to ABA biosynthetic enzymes caused easily dormancy aggravation [37]. Zeaxanthin epoxidase (ZEP) and 9-cis-epoxycarotenoid dioxygenase (NCED) are two kinds of ABA biosynthetic enzymes [12]. It was reported that *ZEP* over-expression increased seed ABA content and enhanced seed dormancy [24]. Furthermore transgenic tomato over-expressing *LeNCED1* delayed germination and increased ABA levels in mature seeds [54]. And expression of bean *PvNCED1* in tobacco resulted in delayed seed germination and an increase of ABA abundance in transgenic seeds [46]. In addition, the abscisicaldehyde oxidase (AAO) involving in the conversion of ABA-aldehyde into ABA was shown to be just highly expressed in vegetative tissues other than reproductive tissues [50].

Ethylene (ET) is another important plant hormone which regulates plant development, tissue growth, seed germination and so on. Ethylene synthesis from 1-Amicocyclopropane-1-carboxillic-acid (ACC) is catalyzed by ACC oxidase (ACO). ACC, the direct precursor for ethylene synthesis, derived from S-adenosyl-Met by ACC synthase (ACS) [5]. Kozarewa et al. [34] found that the thermo-dormancy of lettuce seeds was alleviated by the application of exogenous ethylene or its precursor ACC. And the amount of ethylene increased rapidly during the germination of crop seeds including wheat, corn, soybean and rice [63]. Ethylene could accelerate seed germination by stimulating testa and endosperm rupture [38]. In addition, it was reported that the inhibitory effects of ABA on seed germination could be released by ethylene [22, 29].

Spd participates in tissues development processes via interactions with other plant hormones such as auxins, GA, ABA and ET had been studied [41, 45, 57]. However, information regarding Spd improves seed germination by interacting with other plant hormones is still lacking.

Seed germination begins with water uptake and ends with the emergence of the radical through the surrounding seed tissues [11], which is accompanied by the elongation of the radical and the weakening of the endosperm cap [31, 44]. Hydrogen peroxide (H_2_O_2_) was shown to be associated with endosperm weakening in lettuce seeds. During seed germination, the H_2_O_2_ amount and the peroxidase activity in the endosperm cap of lettuce seed increased rapidly; while the endosperm cap puncture force decreased obviously [65]. However, whether seed germination improvement by Spd is related with the change of H_2_O_2_ is still need further study.

The purpose of this study was to elucidate further the mechanism of Spd promoting seed germination through seed soaking. In present study, the germination test was performed after seed soaking treatments with Spd and inhibitor CHA. The endogenous PAs (Put, Spd and Spm) contents、PAs metabolic emzymes activities and their key biosynthetic genes expressions at the early stage of seed germination (0 and 12 h of seed imbibition) were determined. Meanwhile, the effects of Spd on metabolism of GA, ABA, ET and their biosynthetic genes expressions were examined. H_2_O_2_ and malondialdehyde (MDA) productions in embryo were also analyzed. Illuminating the possible interactions of Spd with phytohormone and peroxide will definitely help us to deeply understand the mechanism of Spd in promotion of sweet corn seed germination.

## Methods

### Materials

Sweet corn (*Zea mays* L.) seeds of Xiantian No.5 were supplied by Sanbei Seed Company, P. R. China, which has been widely planted in southern China due to its high sugar content, better flavor and wide adaptability. Spermidine, cyclohexylamine, 3,3-diaminobenzidine (DAB) and dichlorodihydrofluorescein diacetate (DCFH_2_-DA) were purchased from Aladdin Industrial Inc. Shanghai, P. R. China.

### Seed soaking treatments

Seeds were surface-sterilized for 10 min in 0.1% sodium hypochlorite, and rinsed three times with distilled water. Then 0.9 mM Spd and 16 mM CHA solutions were used for seed soaking at 25 °C, respectively (these concentrations were determined by preliminary experiments); while seeds were soaked in distilled water used as a control.

### Seed germination and seedling characteristics determination

After 12 h of soaking treatments, seed standard germination test was carried out. Three replications each with 100 seeds for each treatment were placed in rolled towels moistened with water, and then were incubated at 25 °C in a growth chamber (Safe, DGX-800E) with 250 μmol · m^−2^ · s^−1^ light intensity and an alternative cycle of 12 h light and 12 h darkness for 7 days.

Seeds were considered as germination when the seed radicle visibly protrudes through the seed coat [10]. The geminated seeds were counted daily for 7 days. Then, the germination energy (GE, the percentage of normal germinated seeds in all tested seeds at the 4th day) and germination percentage (GP, the percentage of normal germinated seeds in all tested seeds at the end of the full test) were calculated on day 4 and day 7, respectively (ISTA, 2010). After 7 days of germination, root length and shoot height were manually measured with a ruler. Root and shoot dry weights were determined after drying at 80 °C for 24 h [13]. In addition, the germination index (GI) was calculated according to GI = ∑ (*Gt*/*Tt*), where *Gt* is the number of the germinated seeds in the *t* day; *Tt* is the time corresponding to *Gt* in days. Seed vigor index (VI) was determined according to the formula VI = GI × seedling dry weight [64].

### The physiological and molecular levels determination in seed embryos

In order to find out the reason why Spd soaking positively improved sweet corn seed germination and vigor, the soaking effect of Spd or CHA on metabolism occurred in seed embryos during imbibition (also known as the early stage of seed germination) was test. The seeds after three soaking treatments were subsequently imbibed in rolled towels moistened with distilled water at 25 °C for 12 h. The embryos of each treatment were freshly excised at 0-h (at the beginning of imbibition) and 12-h imbibition, and those from dry seeds (no soaking) were used as control. All fresh embryos were immediately frozen in liquid nitrogen and stored at −80 °C for further test.

### Endogenous polyamines contents analysis by high performance liquid chromatography (HPLC)

PAs extraction from seed embryos was performed by HPLC according to the method of Flores and Galston [23] with minor modifications. 0.3 g of fresh seed embryos for each treatment was homogenized with 3 ml of 5% (w/v) cold perchloric acid. The homogenates were kept in ice bath for 1 h, and then centrifuged at 23,000 × g for 30 min at 4 °C. The supernatant was transferred to new centrifugal tube and were stored at −80 °C for quantification of PAs. 0.5 ml of supernatant mixed well with 1 ml of 2 mol · L^−1^ NaOH solution and 10 μl of benzoyl chloride and was incubated for 20 min at 37 °C. Then 2 ml of saturated NaCl solution and 2 ml of diethyl ether were added into the above mixture. After centrifugation at 1,500 × g for 5 min at 4 °C, 1 ml of diethyl ether phase was collected, evaporated to dryness under a stream of warm air and redissolved in 100 μl of methanol for test. The above prepared benzoyl-polyamines extract was filtered through a 0.22 μm membrane filter, and then eluted at room temperature through a 6.0 mm × 150 mm, 5 mm particle size reverse-phase (C18) column (Shim-Pack CLC-ODS). PAs peaks were detected by an SPD-20A (Shimadzu) absorbance detector at 254 nm. The mobile phases consisted of methanol and water (64/36, v/v) at a flow rate of 1.0 ml · min^−1^. The polyamine standards (Sigma Chemical Co.) of Put, Spd and Spm were applied for the development of standard curves. These PAs analyses were performed with three biological replications.

### Assay of polyamine biosynthetic enzymes activities

\The activities of ADC, ODC and SAMDC in the seed embryos were determined as described by Matsuda [39] with minor modifications. 0.3 g of fresh seed embryos for each treatment was homogenized in 100 mM potassium phosphate buffer (pH 8.0) containing 0.1 mM phenylmethylsulfonyl fluoride, 1 mM pyridoxal phosphate (PLP), 5 mM dithiothreitol (DTT), 5 mM ethylenediaminetetraacetic acid (EDTA), 25 mM ascorbic acid and 0.1% polyvinylpyrrolidone. The homogenate was centrifuged at 12,000 × g for 40 min at 4 °C and the supernatant dialyzed at 4 °C against 3 mL of 100 mM potassium phosphate buffer (pH 8.0) containing 0.05 mM PLP, 1 mM DTT and 0.1 mM EDTA for 24 h in darkness. The dialyzed extract was used for enzyme assay. The reaction mixture containing 100 mM Tris-HCl buffer (pH 8.5), 1 mM PLP, 5 mM DTT, 5 mM EDTA, 40 mM L-arginine and the dialyzed enzyme extract was used for ADC determination; while the mixture containing 100 mM Tris-HCl buffer (pH 8.0), 1 mM PLP, 5 mM DTT, 5 mM EDTA, 40 mM L-ornithine and the dialyzed enzyme extract was used for ODC determination and the one containing 100 mM potassium phosphate buffer (pH 7.5), 1 mM PLP, 5 mM DTT, 5 mM EDTA, 40 mM S-adenosylmethionine and the dialyzed enzyme extract was used for SAMDC determination. Carbon dioxide production from the each reaction mixture was measured by the conventional Warburg technique at 37 °C for 30 min with gentle agitation. Enzyme activity was expressed in μL · CO_2_ · g^−1^ · DW · min^−1^.

The activity of SPDS in the embryos was determined as described by Yoon [62] with minor modifications. 0.3 g of fresh seed embryos for each treatment was extracted in 100 mM phosphate buffer (pH 8.0) containing 20 mM sodium ascorbate, 1 mM pyridoxal-5-phosphate, 10 mM DTT, 0.1 mM EDTA and 0.1 mM phenylmethanesulfonyl fluoride. After centrifugation, SPDS activity was assayed by incubating an aliquot of the supernatant at 37 °C for 30 min in a reaction mixture consisting of 0.1 M Tris-HCl (pH 8.0), 30 μM Put, 25 μM decarboxylated S-adenosylmethionine, and 20 μM adenine. The reaction product (5-deoxy-5-methylthioadenosyne) was quantified via HPLC (LC-10ATVP, Shimadzu, Japan) and the 1, 7-heptanediamine was used as the internal standard. The ADC, ODC, SAMDC and The SPDS analyses were performed with three biological replications.

### Assays of diamine oxidase and polyamine oxidase activities

Diamine oxidase (DAO) and polyamine oxidase (PAO) activities in the embryos were determined by measuring the generation of H_2_O_2_, a product of the oxidation of polyamines, as described by Su et al. [52] with some modifications. Fresh seed embryos were homogenized in 100 mM potassium phosphate buffer (pH 6.5). The homogenate was centrifuged at 10,000 × g for 20 min at 4 °C and then the supernatant was used for enzyme assay. The reaction mixture contained 2.5 mL of potassium phosphate buffer (100 mM, pH 6.5), 0.2 mL of 4-aminoantipyrine/N, N-dimethylaniline reaction solutions, 0.1 mL of horseradish peroxidase (250 U · mL^−1^) and 0.2 mL of crude enzyme extract. The reaction was initiated by adding 15 mL of Put for DAO determination and adding 7.5 mL of Spd and 7.5 mL of Spm for PAO determination. 0.001 absorbance unit of the change in the optical density at 555 nm · min^−1^ was considered as one unit of enzyme activity. The DAO and PAO analysis were performed with three biological replications.

### Gibberellins and abscisic acid concentrations analysis by HPLC

0.5–1 g of fresh seed embryos for each treatment was homogenized with 4 ml of cold acetonitrile. The homogenates were kept at 4 °C for 12 h and then centrifuged at 23,000 × g for 10 min at 4 °C. The supernatant was transferred to new centrifugal tube and mixed with 3 ml of phosphate buffer solution (0.1 M). The mixture was firstly kept at −80 °C for 30 min and thawed adequately at 4 °C. Then after the addition of 1 ml of hydrochloric acid (0.5 mM), the mixture was extracted by 5 ml of ethyl acetate for three times. 12 ml of the ethyl acetate phase was collected after extract centrifuged at 1500 × g for 5 min at 4 °C, and then it was evaporated to dryness under a stream of warm air, and redissolved in 1 ml of mobile phases which consisted of methanol and water (1/1, v/v).

The above prepared extract was filtered through a 0.22 mm membrane filter, and then eluted at room temperature through a 6.0 mm × 150 mm, 5 mm particle size reverse-phase (C18) column (Shim-Pack CLC-ODS). GA_3_ and ABA peaks were detected by an SPD-20A (Shimadzu) absorbance detector at 254 nm. The mobile phases consisted of methanol and water (1/1, v/v) at a flow rate of 0.8 ml · min^−1^. The standards of GA_3_ and ABA (Sigma Chemical Company) were used for the development of standard curves. The GA and ABA analysis were performed with three biological replications.

### Ethylene production analysis by gas chromatography (GC)

The ethylene production (nmol · g^−1^ · h^−1^ · FW) in seed embryo was measured by enclosing 10 seeds in 50 ml of air-tight container for 3 h at 25 °C. 1 ml of the headspace gas was took out and injected into a gas chromatograph (model Agilent, 6890 N, USA) fitted with a flame ionization detector and an activated alumina column. The measurement conditions were as follows: chromatograph column, HP-55% phenyl methyl siloxane, 30 m capillary alumina column (Agilent 19091 J-413); the temperature of column and detector was 80 °C and 150 °C, respectively; the flow rate of carrier gas N_2_ was 40 ml min^−1^and the hydrogen pressure was 0.6 kg · cm^−2^. The ethylene analysis was performed with three biological replications.

### Hydrogen peroxide quantification analysis

H_2_O_2_ content (nmol · g · FW^−1^) in the embryos was colorimetrically measured as described by Huang et al. [30] with minor modifications. 0.3 g of fresh seed embryos for each treatment was homogenized with 3 ml of cold acetone. The homogenate was centrifuged at 12,000 rpm for 15 min. 1 ml of supernatant was mixed with 0.1 ml of 5% titanium sulphate in concentrated HCl, followed by adding 0.2 ml of aqueous NH_3_ (25%) to precipitate the peroxide-titanium complex. After centrifugation at 12,000 rpm for 15 min, the precipitate was solubilized in 3 ml of 2 mM H_2_SO_4_. The absorbance of the obtained solution was read at 415 nm. The standard response curve was prepared with a known concentration of H_2_O_2_ using the same method as described above. The H_2_O_2_ analysis was performed with three biological replications.

### Histochemical and fluorescence localization of hydrogen peroxide

Histochemical localization of H_2_O_2_ in seed embryos was carried out by DAB (3, 3-diaminobenzidine) staining methods as described by Thordal-Christensen et al. [55] with minor modifications. The embryos were incubated in 1 mg · ml^−1^ DAB-HCl (pH 3.8) at room temperature for 5 h, and then they were washed with distilled water and observed. Fluorescence localization of H_2_O_2_ in seed embryos was carried out by DCFH_2_-DA (2, 7-Dichlorodihydrofluorescein diacetate) staining method as described by Sakamoto et al. [48] with minor modifications [47]. The tip of radical was stained with 25 μM DCFH_2_-DA for 5 min, rinsed with distilled water, and then observed under an FV1000 co-focal laser scanning microscope (Olympus).

### Malondialdehyde concentration measurement

Malondialdehyde (MDA) concentration in the embryo was determined as described by Cao et al. [14] with minor modifications. Briefly, 0.3 g of fresh seed embryos were ground in 8 ml of 0.05 M sodium phosphate buffer (pH 7.8) and centrifugated at 10,000 × g for 15 min. A 1.5 ml of supernatant was transferred into 2.5 ml of 5% trichloroacetic acid (TCA) solution containing 0.5% (w/v) thiobarbituric acid (TBA), which was maintained at 100 °C water bath for 15 min and then cooled immediately. After centrifugation at 1,800 × g for 10 min, the absorption of the supernatant was measured spectrophotometrically at 532 nm and 600 nm. MDA concentration was calculated using an extinction coefficient of 155 mM^−1^ · cm^−1^ and expressed as nmol · g^−1^ · FW. The MDA analysis was performed with three biological replications.

### RNA isolation and quantitative RT-PCR of metabolism-related genes

Total RNA was extracted from seed embryos of each sample with RNeasy Mini Kit (HuaYueYang, Beijing, China) according to the manufacturer’s protocol, and was used for quantitative RT-PCR analysis after RNA concentration and purity determination by scanning UV spectroscopy.

The PCR amplifications were performed with gene-specific primers (shown in Table 1). The 18 s rRNA gene was used as an internal control. According to the established protocol, the cDNA was amplified using SYBR-Green PCR Master kit (Applied Biosystems, Foster City, CA, USA) containing a AmpliTaq Gold polymerase on a iCycler (BioRad, Munich, Germany). Real-time PCR efficiency was estimated via a calibration dilution curve and slope calculation. Expression levels were determined as the number of cycles needed for the amplification to reach a threshold fixed in the exponential phase of the PCR (CT). Relative expression values were normalized respect to the sample of control at 0 h of imbibition time. Real-time PCR analysis was performed with three biological replications, and each was made in three technical replicates.

**Table 1:** Real-time PCR primers for genes expressions detection

Gene name	Accession no.	Primer sequence
*18s rRNA*	NC_008332.1	F: 5′- ACATGCGCCTAAGGAGAAATAG -3′ R: 5′- ACCTCCATGCTCACTGGTACTT -3′
*ZmADC1*	NM001323076	F: 5′- GCTACGGCTCAAGGTACCAG-3′ R: 5′- CCGAACTCCACAATGTCCTC-3′
*ZmODC*	NM 001148682	F: 5′- GCGCCTACTCCACAGGTTC -3′ R: 5′- CGTAGATCTTAATCTCCGACGTG-3′
*ZmSPDS*	NM 001155838	F: 5′- TGTTCAATTCCATCCCCTAAA -3′ R: 5′- TCCACTGAACTGTGTCTGGAA-3′
*ZmSAMDC2*	NM 001112243	F: 5′- TGTGGCTACTCCATGAATGC-3′ R: 5′- CGTAACTGGCGTAGCTGAAA-3′
*ZmSPMS*	AY730048	F: 5′- ATCCTCGTGTCCGACTTCA-3′ R: 5′- CATCATATTTTCCTTCAGGGAGA-3′
*ZmGA3ox1*	NM 001279524	F: 5′- CACCATGCATCTCAACTGGTA-3′ R: 5′- GAAGGTGAAGAAGCCCGAGT -3′
*ZmGA20ox4*	NM 001156050	F: 5′- GAGAGGTTCTCCATGCCCTA-3′ R: 5′- AAGAAGTCGCCCCAGTTGTA -3′
*ZmGA2ox*	NM 001158585	F: 5′- GAGAGGTGTGGGTTCAGGTG -3′ R: 5′- CGAGCTTGCAATTACCCTGT -3′
*ZmNCED1*	XM 008672757	F: 5′- GCACCGTGGAGAAGTTCG -3′ R: 5′- ACAGCACGTACCCGTCATC -3′
*ZmZEP*	NM 001157971	F: 5′- CAGCACGGCATAACACCTC -3′ R: 5′- GCGAAAGAGAAGACGTTGGA-3′
*ZmAAO*	NM 001111838	F: 5′- CTGGAATATCACCAGATGTGAGTC-3′ R: 5′- TGCCCCAGTTGTACTTTTTGA-3′
*ZmACS*	NM 001152929	F: 5′- GACGCCTTATTACCCAGCTTT -3′ R: 5′- ATGGGCAGCAGCTTCACT -3′
*ZmACO1*	NM 001136755	F: 5′- ACCGCTACAGGCAGGTGAT -3′ R: 5′- CTCCAGGCCCAGGTTCTC -3′

### Statistical analysis

The data were subjected to an analysis of variance (ANOVA) on the Statistical Analysis System (SAS) software. The multiple comparison for mean values were performed by Tukey’S Honestly Significant Difference (HSD) test (*P* < 0.05). Before ANOVA, the data of percentage were transformed according to *y* = arcsin [sqrt (*x*/100)].

## Results

### Effects of exogenous spermidine and cyclohexylamine on seed germination and seedling characteristics

**Fig. 1:**
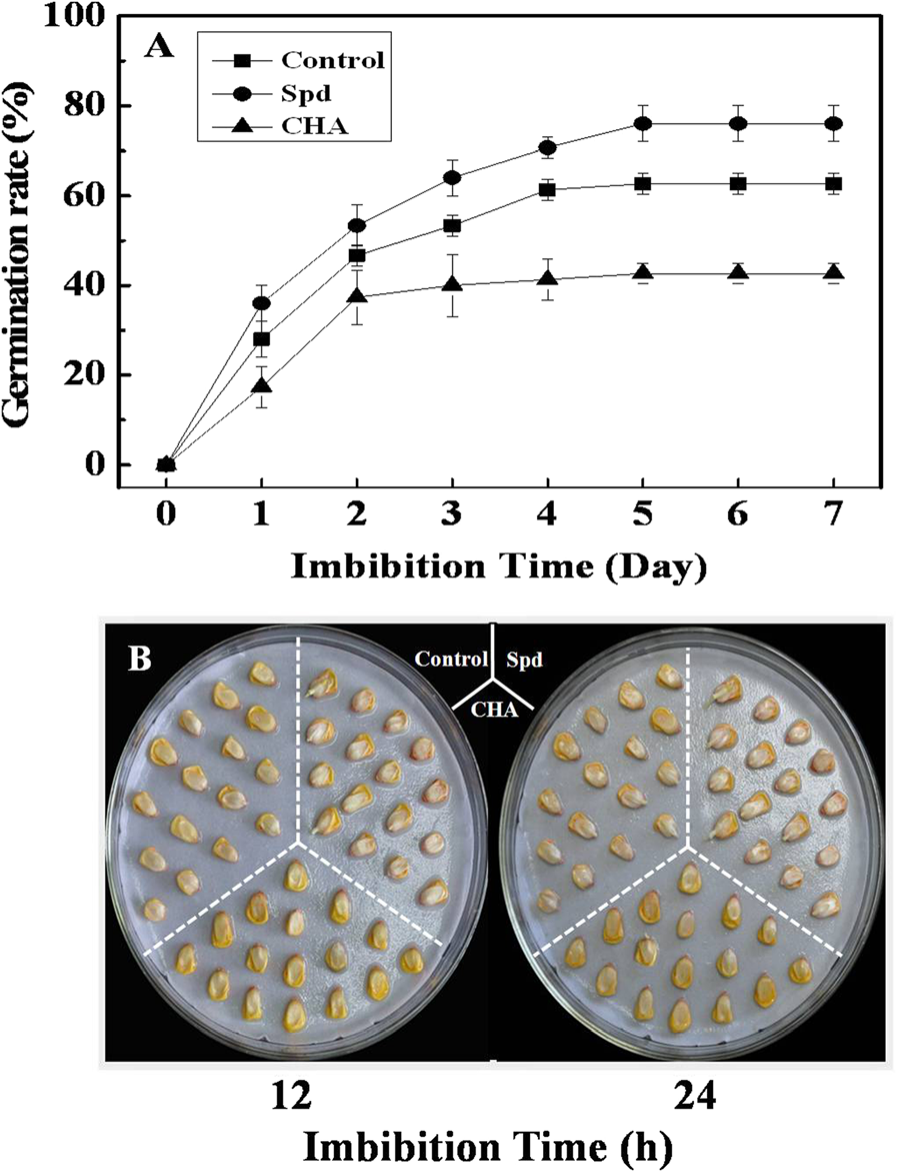
Effects of soaking treatments on sweet corn seed germination. **a** Time courses of seed germination under 25 °C. Control, seeds soaking with distilled water; Spd, seeds soaking with spermidine; CHA, seeds soaking with cyclohexylamine. Results are representative of three independent experiments. Vertical bars indicate ± SE of mean (*n* = 3). **b** Seed germination of sweet corn. Seeds were imbibed in rolled towels moistened with water at 25 °C for 12 or 24 h, respectively

Seeds soaked with Spd germinated much faster than the control and CHA treatment. Seed germination percentage of Spd treatment had reached 38% after 24 h of imbibition which was higher than 23% of control and 18% of CHA (Fig. 1a). After 7 days of germination, Spd treatment showed significant higher germination energy, germination percentage, germination index and vigor index compared with control (Table 2). While the germination energy, germination percentage, germination index and vigor index of CHA treatment were significantly lower than those of the control and Spd treatment. Seeds soaked in Spd germinated noticeably faster than those of control and CHA after 24 h of imbibition (Fig. 1b).

**Table 2:** Effects of seed soaking treatments with spermidine (Spd) or cyclohexylamine (CHA) for 12 h on seed germination energy (GE), germination percentage (GP), germination index (GI) and vigor index (VI) of sweet corn, Xiantian No.5 under normal temperature (25 °C)

Treatment	GE (%)	GP (%)	GI	VI
Control	61 ± 2.31b	63 ± 2.31b	10.46 ± 0.71b	3.48 ± 0.39b
Spd	71 ± 2.31a	76 ± 1.02a	12.7 ± 0.71a	5.63 ± 0.44a
CHA	41 ± 4.62c	43 ± 2.31c	7.21 ± 1.10c	1.81 ± 0.29c

Values were mean ± SE (*n* = 3). Different small letter (s) following the values indicated significant difference (*p* < 0.05, Tukey’s HSD) among treatments. Control, seeds soaked in distill water as a control; Spd, seeds soaked in 0.9 mM spermidine solution; CHA, seeds soaked in 16 mM cyclohexylamine solution

Compared with the control, seedling growth were improved by exogenous Spd, of which shoot dry weight, root dry weight and shoot height reached significant levels (Table 3, Additional file 1). On the contrary, CHA treatment significantly decreased shoot dry weight and root dry weight. And the shoot height and root length of CHA treatment were also obviously lower than those of the control (Table 3, Additional file 1).

**Table 3:** Effects of seed soaking treatments with spermidine (Spd) or cyclohexylamine (CHA) for 12 h on seedling shoot dry weight, root dry weight, shoot height and root length of sweet corn, Xiantian No.5 under normal temperature (25 °C)

Treatment	Shoot dry weight (g/10seedlings)	Root dry weight (g/10seedlings)	Shoot height (cm/seedling)	Root length (cm/seedling)
Control	0.20 ± 0.011b	0.14 ± 0.004b	9.96 ± 0.249b	10.23 ± 0.206a
Spd	0.26 ± 0.004a	0.18 ± 0.010a	12.11 ± 0.755a	10.99 ± 1.235a
CHA	0.16 ± 0.009c	0.09 ± 0.004c	8.93 ± 0.487b	9.30 ± 1.107a

Values were mean ± SE (*n* = 3). Different small letter (s) following the values indicated significant difference (*p* < 0.05, Tukey’s HSD) among treatments. For other explanation please see Table 2

### Effects of spermidine and cyclohexylamine on polyamines contents and polyamine-related genes expressions

**Fig. 2:**
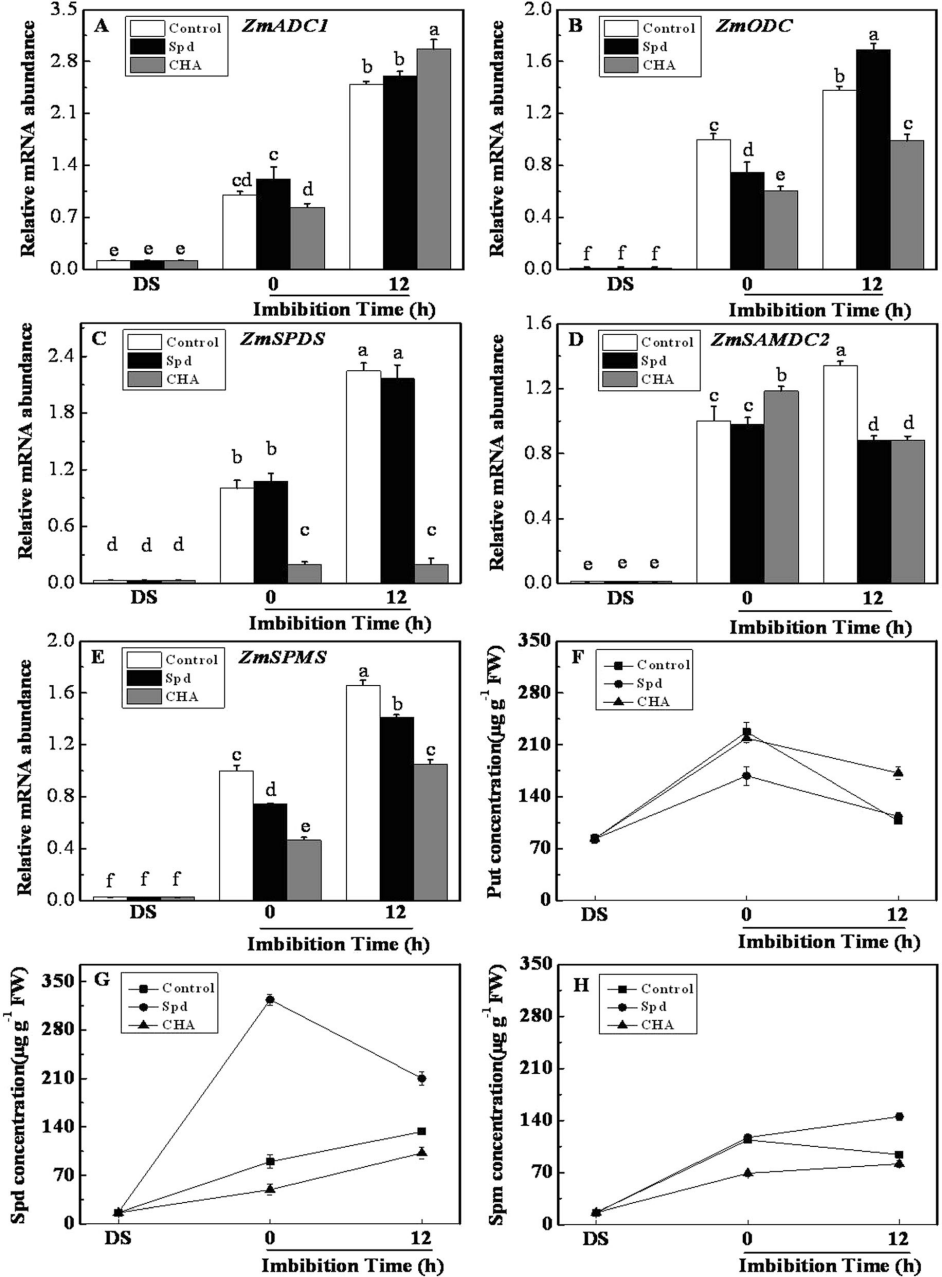
Effects of soaking treatments on polyamine genes expressions and contents in sweet corn seed embryos. Relative mRNA abundances of *ZmADC1* (**a**), *ZmODC* (**b**), *ZmSPDS* (**c**), *ZmSAMDC2* (**d**), *ZmSPMS* (**e**) and changes of Put (**f**), Spd (**g**) and Spm (**h**) concentrations in sweet corn seed embryos. Control, seeds soaking with distilled water; Spd, seeds soaking with spermidine; CHA, seeds soaking with cyclohexylamine; DS, the seed embryos isolated from dry seeds (before soaking treatment); Put, putrescine; Spd, spermidine; Spm, spermine. Results are representative of three independent experiments. Different small letter(s) on the top of the bars indicate significant differences (*p* < 0.05, Tukey’s HSD) among treatments. Error bars denote SE (*n* = 3) of biological replicates within an experiment

The endogenous Spd concentration of sweet corn seed was significantly augmented by exogenous Spd as compared with the control and CHA treatment. Even after 12 h of imbibition, it was still nearly two times of those in control and CHA (Fig. 2g). The Spm concentration increased during seed imbibition as a response to Spd application, and reached significant level as compared with the control and CHA treatment after 12 h of imbibition (Fig. 2h). However, the Put concentration was always significantly lower in Spd treatment than that in CHA from 0 to 12 h of imbibition (Fig. 2f). The expressions of polyamines, GA, ABA and ethylene metabolism-related genes (including gene family members) which were reported presently in many studies on maize were all analyzed (Additional file 2, Additional file 3), but only the genes which were regulated differently by three treatments in the process of seed imbibition were selected for detailed showing in this study. Figure 2 showed that the transcript levels of polyamines biosynthesis genes including *ZmADC1*, *ZmODC*, *ZmSPDS, ZmSPMS and ZmSAMDC2* increased rapidly after seed soaked in distilled water during seed imbibition. The similar results with Spd and CHA treatments were observed in *ZmADC1* and *ZmODC* expressions (Fig. 2a, b). However it was noteworthy that at 12-h imbibition, as compared with the control (soaking in distilled water), Spd increased the transcript level of *ZmODC* but made no significant effect on the *ZmADC1* transcript level; while CHA significantly decreased and increased the transcript level of *ZmODC and ZmADC1*, respectively. As compared with the control, the transcript level of *ZmSPDS* in seed significantly decreased and kept lowest level during seed imbibition after exogenous inhibitor CHA treatment; while there were no significant differences in *ZmSPDS* transcript level between Spd treatment and the control (Fig. 2c). In addition, the expressions of *ZmSAMDC2* and *ZmSPMS* were significantly inhibited by Spd and CHA at 12-h imbibition (Fig. 2d, e). In general, the exogenous CHA inhibited Spd biosynthesis by inhibiting the expression of *ZmSAMDC2* and *ZmSPDS* and exogenous Spd made no significant difference with the biosynthesis of Spd.

### Effects of spermidine and cyclohexylamine on polyamine anabolic and catabolic enzymes activities

**Fig. 3:**
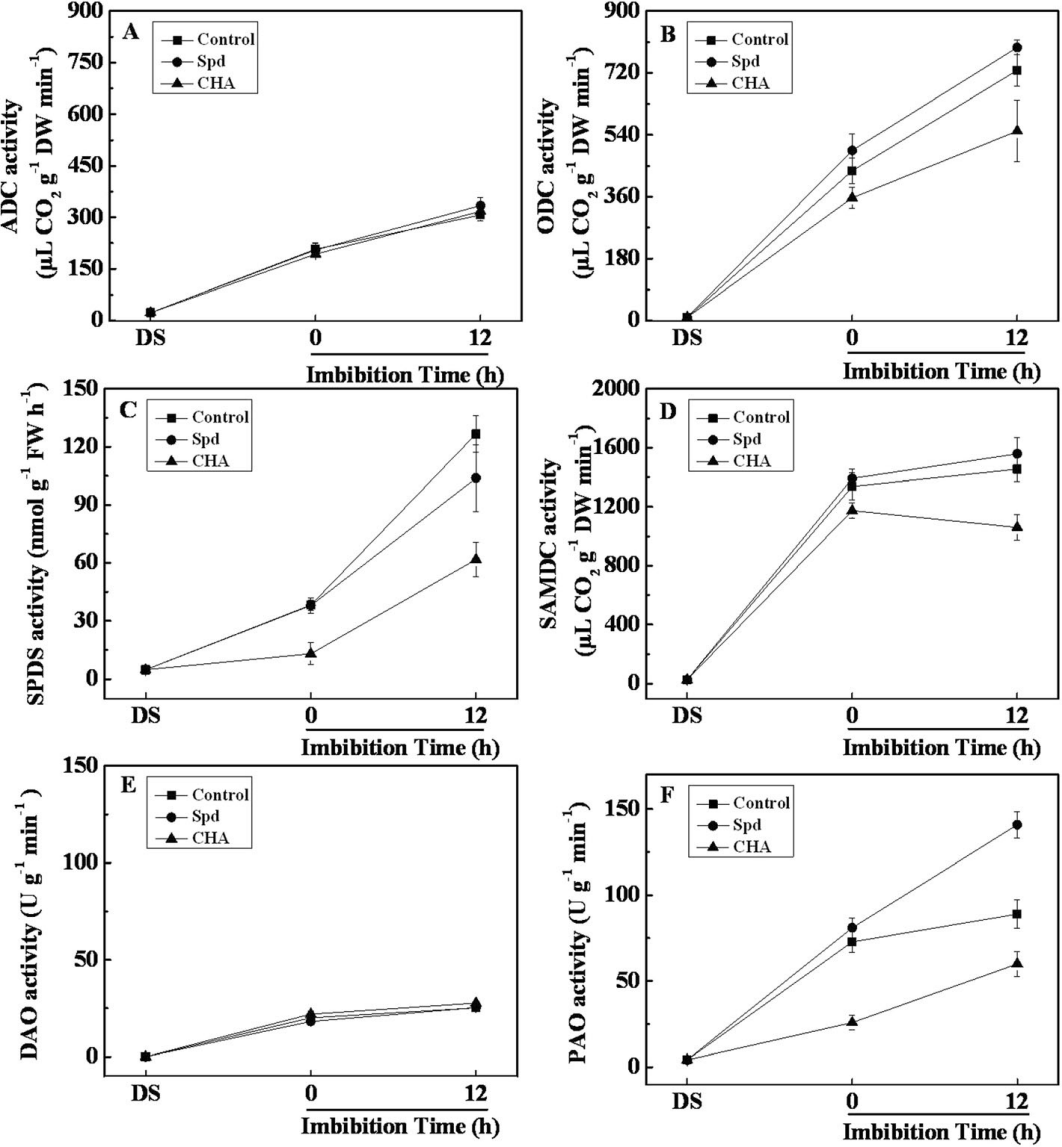
Effects of soaking treatments on polyamine metabolic enzymes activities in sweet corn seed embryos. Activities of arginine decarboxylase (**a**, ADC), ornithine decarboxylase (**b**, ODC), spermidine synthase (**c**, SPDS), S-adenosylmethionine decarboxylase (**d**, SAMDC), diamine oxidase (**e**, DAO) and polyamine oxidase (**f**, PAO) in sweet corn seed embryos during seed imbibition. Control, seeds soaking with distilled water; Spd, seeds soaking with spermidine; CHA, seeds soaking with cyclohexylamine; DS, the seed embryos isolated from dry seeds (before soaking treatment). Results are representative of three independent experiments. Error bars denote SE (*n* = 3) of biological replicates within an experiment

Several polyamine anabolic and catabolic enzymes activities in seed embryos were all promoted by water soaking (Fig. 3). In comparison with control, ODC activity was increased by Spd at 0 and 12 h of imbibition, which however was obviously decreased by CHA (Fig. 3b). No notable differences in ADC activity were found in seed embryos treated with control, Spd and CHA (Fig. 3a). CHA treatment caused obviously decreases in SPDS and SAMDC activities (Fig. 3c, d). However, except SPDS activity at 12-h imbibition, there were no remarkable differences in SPDS and SAMDC activities between exogenous Spd and control (Fig. 3c).

For polyamine oxidase, exogenous Spd caused a significant increase in PAO activity at 12-h imbibition compared with control, while CHA decreased obviously PAO activity during seed imbibition (Fig. 3f). However, the DAO activity was not affected by Spd or CHA application in comparison with control (Fig. 3e).

### Effects of spermidine and cyclohexylamine on GA_3_ contents and GA-related genes expressions

**Fig. 4:**
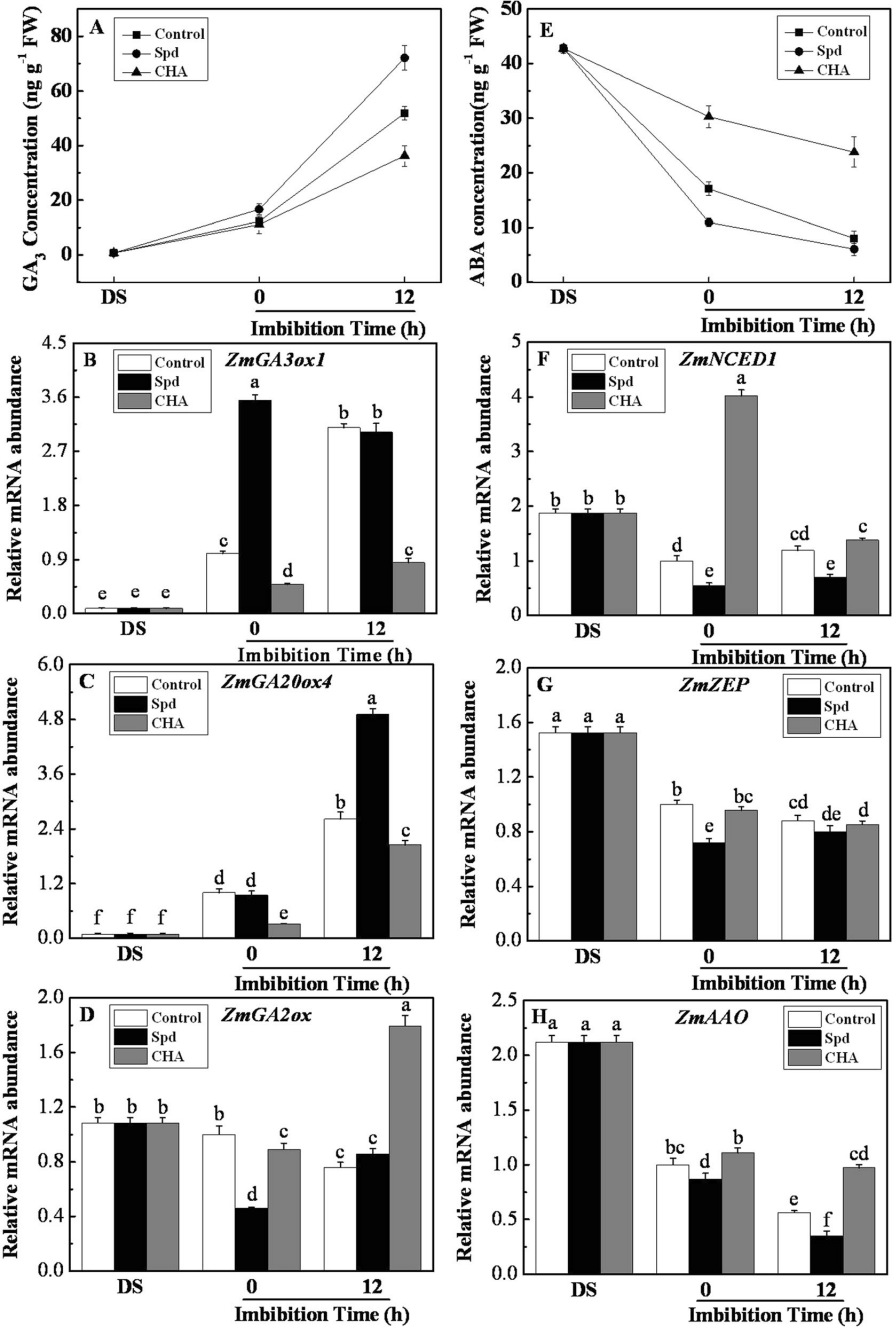
Effects of soaking treatments on GA_3_ and ABA contents and metabolism related genes expressions in sweet corn seed embryos. Changes in gibberellin (**a**, GA_3_) concentration, abscisic acid (**e**, ABA) concentration and relative mRNA abundances of *ZmGA3ox1* (**b**), *ZmGA20ox4* (**c**), *ZmGA2ox* (**d**), *ZmNCED1* (**f**), *ZmZEP* (**g**) and *ZmAAO* (H) in seed embryos. Control, soaking with distilled water; Spd, soaking with spermidine; CHA, soaking with cyclohexylamine; DS, the seed embryos isolated from dry seeds (before soaking treatment). Results are representative of three independent experiments. Error bars denote SE (*n* = 3) of biological replicates within an experiment. Different small letter (s) on the top of the bars indicate significant difference (*p* < 0.05, Tukey’s HSD) among treatments

In comparison with the control, exogenous Spd significantly elevated GA_3_ levels in the process of seed germination, while CHA significantly decreased GA_3_ levels (Fig. 4a).

The transcript level of *ZmGA3ox1* and *ZmGA20ox4* increased rapidly during seed imbibition after seed treated with distill water (Fig. 4b, c). Exogenous Spd significantly increased *ZmGA3ox1* expression at 0 h of imbibition and *ZmGA20ox4* expression at 12 h of imbibition as compared with the control; while CHA decreased significantly the expressions of *ZmGA3ox1* and *ZmGA20ox4* in the process of seed imbibition (Fig. 4b, c). The expression of *ZmGA2ox* had the highest level in dried seed, and decreased significantly during seed imbibition of control (Fig. 4d). Although the application of Spd and CHA significantly decreased *ZmGA2ox* expression at 0-h imbibition compared with control, CHA increased significantly *ZmGA2ox* expression than Spd did at 12-h imbibition (Fig. 4d).

### Effects of exogenous spermidine and cyclohexylamine on ABA contents and ABA-related genes expressions

As compared with the control and CHA treatment, exogenous Spd significantly lowered ABA levels at 0 h of imbibition. However, CHA significantly elevated ABA levels at 0 and 12 h of imbibition (Fig. 4e). The transcription of *ZmNCED1*, *ZmAAO* and *ZmZEP* had the highest levels in dried seed embryo, and decreased rapidly during seed imbibition after seed treated with distill water (Fig. 4f, g, h).

Exogenous Spd significantly decreased *ZmNCED1* and *ZmAAO* levels at 0 and 12 h of imbibition compared with the control; while CHA significantly increased *ZmNCED1* at 0 and 12 h of imbibition and increased *ZmAAO* at 12-h imbibition (Fig. 4f, h). The application of CHA made no difference on *ZmZEP* expression during seed imbibition as compared with the control, however exogenous Spd significantly decreased *ZmZEP* expression at 0-h imbibition (Fig. 4g).

### Effects of exogenous spermidine and cyclohexylamine on ethylene contents and ethylene-related genes expressions

**Fig. 5:**
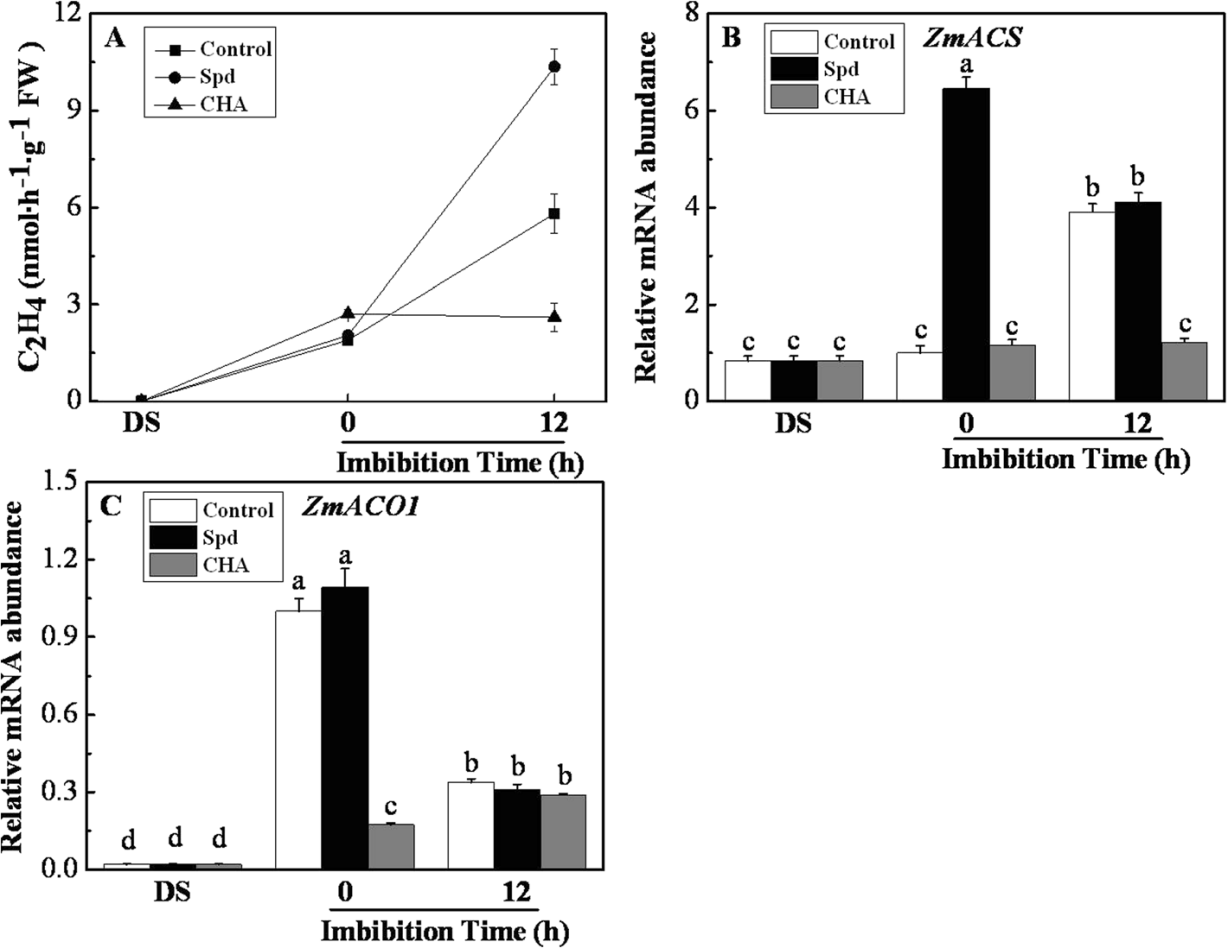
Effects of soaking treatments on ethylene content and metabolism related genes expressions in sweet corn seed embryos. Change in the ethylene (**a**) level and relative mRNA abundances of *ZmACS* (**b**) and *ZmACO1* (**c**) in seed embryos. Control, soaking with distilled water; Spd, soaking with spermidine; CHA, soaking with cyclohexylamine; DS, the seed embryos isolated from dry seeds (before soaking treatment). Results are representative of three independent experiments. Error bars denote SE (*n* = 3) of biological replicates within an experiment. Different small letter (s) on the top of the bars indicate significant difference (*p* < 0.05, Tukey’s HSD) among treatments

The process of seed imbibition was accompanied by the increasing of ethylene production in the control (Fig. 5a). There were no significant differences on C_2_H_4_ content after seed soaked in Spd and CHA (at 0 h of imbibition) compared with control. However, at 12 h of imbibition, ethylene increased remarkably in the seeds treated with exogenous Spd; while it significantly decreased after CHA treatment (Fig. 5a). Exogenous Spd significantly increased the expression of *ZmACS* at 0 h of imbibition compared with control and CHA (Fig. 5b). During seed imbibition, the *ZmACS* expression obviously decreased and increased in Spd treatment and the control, respectively, and finally there were no significant difference between Spd and the control at 12 h of imbibition. The *ZmACS* expression always kept at a low level in CHA treatment. In addition, Spd had no effect on *ZmACO1* expression compared with the control in the process of seed imbibition (Fig. 5c). However, the expression of *ZmACO1* was significantly lower in CHA than that in the control, and maintained a low level during seed imbibition (Fig. 5c).

### Effects of exogenous spermidine and cyclohexylamine on H_2_O_2_ and MDA contents

**Fig. 6:**
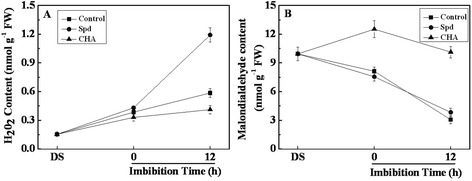
Effects of soaking treatments on H_2_O_2_ and MDA contents in sweet corn seed embryos. Changes in the hydrogen peroxide (**a**, H_2_O_2_) and malondialdehyde (**b**, MDA) contents in seed embryos. Control, soaking with distilled water; Spd, soaking with spermidine; CHA, soaking with cyclohexylamine; DS, the seed embryos isolated from dry seeds (before soaking treatment). Results are representative of three independent experiments. Error bars denote SE (*n* = 3) of biological replicates within an experiment

H_2_O_2_ content in seed embryos increased during seed imbibition in all three treatments (Fig. 6a). After 12-h imbibition, Spd application dramatically increased H_2_O_2_ content by twofold compared with the control; while CHA significantly decreased H_2_O_2_ content (Fig. 6a).

The MDA content in seed embryo decreased remarkably during seed imbibition in Spd treatment and control, and no obvious difference between Spd and control was detected (Fig. 6b). However, in comparison with control, the MDA content increased by 1.5-fold and 3.3-fold in CHA treated seed embryos at 0 and 12 h of imbibition respectively.

**Fig. 7:**
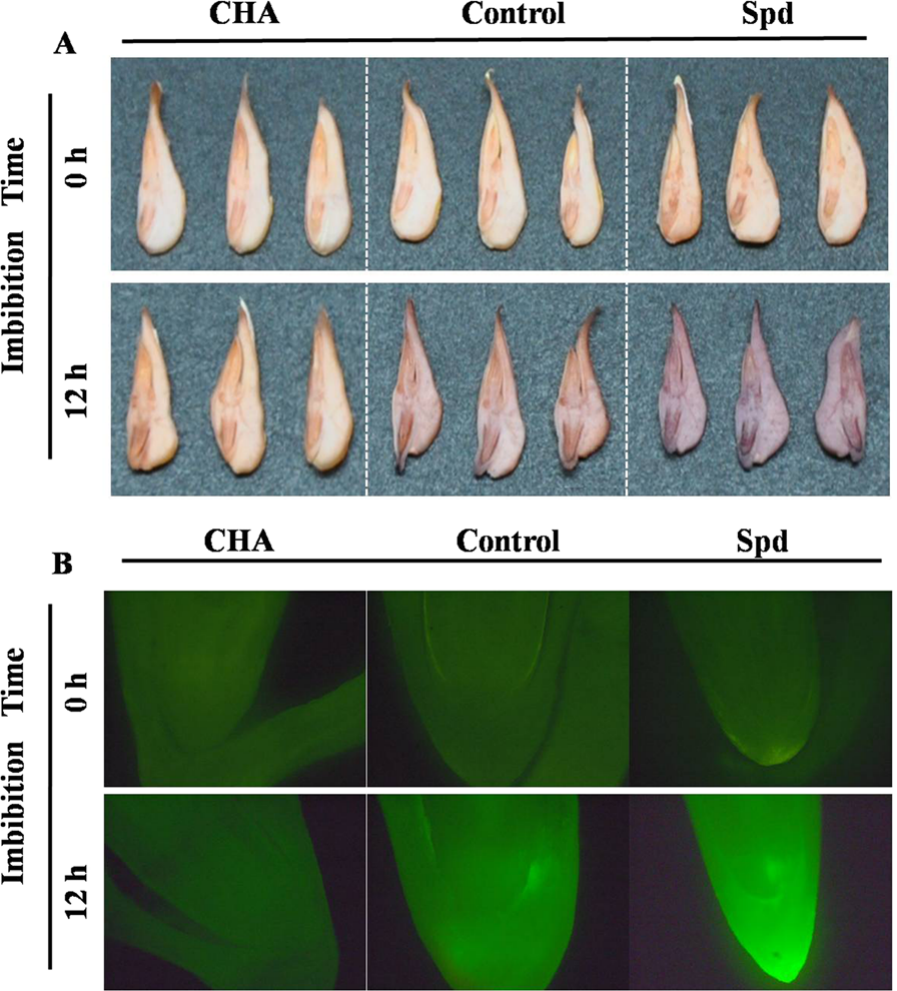
Effects of soaking treatments on the localization of H_2_O_2_ in sweet corn seed embryos and radical tip. **a** Histochemical localization of hydrogen peroxide (H_2_O_2_) in seed embryos. Control, soaking with distilled water; Spd, soaking with spermidine; CHA, soaking with cyclohexylamine. The seed embryos were isolated from seeds after 0 and 12 h of imbibition in rolled towels moistened with water at 25 °C. Bars, 2 mm. **b** Fluorescence localization of H_2_O_2_ in seed radical tip. The seed radicals were isolated from seed embryos after 0 and 12 h of imbibition. The presence of H_2_O_2_ was shown by green fluorescence. Bars, 50 μm

In addition, the accumulation of H_2_O_2_ in embryo was detected visually by DAB staining. The radical tip of seed imbibed in water (control) showed strongest intensity (Fig. 7a). Although the intensity of the staining in the plumule and radical had no difference among control, Spd and CHA treatments at 0-h imbibition, it dramatically increased by Spd application and obviously decreased by CHA treatment compared with the control at 12-h imbibition.

The similar results were found in the fluorescence localization of H_2_O_2_ in radical tip (Fig. 7b). It showed that the fluorescence in radical tip (indicating H_2_O_2_ intensity) treated by Spd was much brighter than those treated by the control and CHA treatment at 12 h of imbibition.

## Discussion

According to the results, Spd soaking treatment not only accelerated seed germination of sweet corn but also significantly enhanced seed vigor as compared with the control. On the contrary, exogenous CHA significantly inhibited seed germination and seed vigor. CHA is the inhibitor of spermidine synthase (SPDS), which directly inhibits the SPDS activity and led to the depression of Spd content. The depression of Spd content in seed embryo induced by CHA might be an important reason for the decrease of seed vigor. Therefore, Spd was believed to play an important role in physiological metabolism during sweet corn seed germination. This is consistent with the results reported by Du et al. [20], who found that seed soaking with appropriate concentration of Spd could promote seed germination of ordinary corn. Besides, exogenous Spd was shown to improve rice seed vigor under salt stress [58] and enhance clover seed germination under low temperature [36], which indicated that Spd definitely made a positive effect on seed germination.

To test the effects of soaking treatments with exogenous Spd and CHA on the metabolism of endogenous polyamines in embryos, the endogenous polyamines content, the transcript levels of polyamines biosynthesis genes and polyamines metabolic enzymes activity were determined. It showed that the presence of Spd increased the endogenous Spd and Spm levels of sweet corn seeds, however made no significantly difference on *ZmADC1* and *ZmSPDS* expressions, and even decreased *ZmSAMDC2* and *ZmSPMS* expressions during seed germination. Also the transcriptional levels of *ZmADC2* and *ZmSAMDC1* did not respond to Spd or CHA treatment (Additional file 2). In similar with the results of genes expressions, enzyme activity analysis revealed that Spd application did not cause an obvious increase in PAs biosynthetic enzymes activities, even decreased the SPDS activity at 12 h of imbibition. Thus it was proposed that the increased endogenous Spd levels in Spd-treated seed embryos might mainly from the exogenous Spd permeation other than new Spd synthesis. In addition, the present study suggested that the depression of Spd and Spm contents in CHA-treated seed embryos mainly because exogenous inhibitor CHA significantly decreased *ZmSPDS* expression and even *ZmODC* and *ZmSAMDC2* transcripts, which leading to the decreased activities of SPDS, ODC and SAMDC. In discordance with this phenomenon, Scoccianti et al. [49] indicated that 5 mM CHA treatment resulted in a dramatic increase in the level of Put but has little or no effect on the levels of Spd and Spm in germinating pollen of kiwifruit. Therefore, it was proposed that the influence of CHA upon PAs differs depending on species, organs, physiological processes and even CHA concentration.

Gibberellins are a family of plant hormones controlling many aspects of plant growth and development including stem elongation and seed germination [52, 53]. And the interaction between PAs and GAs had been studied preliminarily in some species such as Arabidopsis and tomato [2, 27, 32]. Alcázar et al. [2] indicated that the high endogenous Spd/Spm concentrations in the *35S:AtADC2* Arabidopsis plants were accompanied by a significant drop in *AtGA3ox3* and *AtGA20ox1* expression as well as reduced GA production. In addition, significantly increased *GA2ox* expression and reduced GA content was observed in *E8:ySAMDC* transgenic tomato fruits, which accumulated three to four times in Spd concentration compared with control [32]. However, in our study, we found that the application of Spd significantly increased the GA content of embryo via increasing the transcript levels of GA biosynthesis genes *ZmGA3ox1* and *ZmGA20ox4* in the process of seed imbibition. On the contrary, that the exogenous CHA decreased the expressions of *ZmGA3ox1*, *ZmGA20ox4* and increased simultaneously the expression of *ZmGA2ox* dramatically restricted GA biosynthesis. However, the expressions of *ZmGA3ox2*, *ZmGA20ox1*, *ZmGA20ox2* and *ZmGA20ox3* did not respond to Spd or CHA treatment (Additional file 2). The results indicated that the increase of GA content in seed embryo of sweet corn induced by Spd might be attributed mainly to the increase in both *ZmGA3ox1* and *ZmGA20ox4* expressions instead of their other family members. And the synthesis of GA in seed embryo might be down-regulated by exogenous CHA in transcriptional level. Therefore, it was proposed that the influence of Spd upon GA differs depending on species, organs and physiological processes.

The interaction between ABA and PAs during seed maturation and germination had also been studied in detail [4, 6, 26]. ABA treatment obviously enhanced Put and Spm but decreased Spd content in wheat seeds [33]. Spm relieved polyethylene glycol (PEG)-induced osmotic stress by down-regulating ABA and antioxidant levels in soybean pods and seeds [47]. Application of Spd did not alter ABA levels in radish seedlings under normal growth condition [17] or chromium stress [16], but reduced ABA accumulation induced by copper stress in radish seedlings [17]. Spd accumulation in *35S:AtSAMDC1* Arabidopsis leaves inhibited expression of *ALDEDHYE OXIDASE* (*AAO2*) [27]; while enhanced levels of Spd and Spm in transgenic *E8:ySAMDC* tomato fruits decreased *AAO4* transcripts and increased abscisic acid 8-hydroxylase 3 transcripts [32].

In agreement with the reports above, our data showed that exogenous Spd significantly decreased *ZmAAO* transcripts and the expression of *ZmNCED1*, which leading to the decrease of ABA levels in Spd-treated seeds. On the contrary, the expressions of *ZmNECD1* and *ZmAAO* increased obviously after application of exogenous CHA, which was accompanied by the increase of ABA content during seed germination. However exogenous Spd or CHA made no significant effect on the expressions of *ZmNCED2* and *ZmNCED3*. These results suggested that Spd improved seed germination and seed vigor to some extent was closely associated with the regulation of GA and ABA metabolism at their transcriptional levels. And gene family members had different responses to Spd stimulation.

Ethylene is wildly involved in leaf epinasty, flower fading, abscission, fruit ripening and seed germination [15]. It was demonstrated that exogenous application of PAs inhibits ethylene production in plant tissues, and that ethylene inhibits the enzymes activities involved in PA biosynthesis pathway, which might be due to the SAM was the common substrate for ethylene, Spd and Spm biosynthesis [15, 51]. However, a much higher ethylene production was observed in *E8:ySAMDC* transgenic tomato fruit, which simultaneously accumulated two to three times in Spd/Spm concentration compared with the control [41]. It was suggested that availability of SAM was not rate limiting for the biosynthesis of either ethylene or Spd/Spm and that these two pathways could run simultaneously.

In the present study, we found an increase in the ethylene level in embryo during seed germination and in response to exogenous Spd; meanwhile, the ethylene production was down-regulated by exogenous CHA. Additionally, the data showed that the transcriptional level of *ZmACS* was up-regulated by Spd and down-regulated by CHA and the transcriptional level of *ZmACO1-5* (Additional file 2) was unaffected. In generally, higher ethylene levels as a consequence of higher expression of *ZmACS* in embryo induced by exogenous Spd was closely associated with the seed germination improvement in corn. A similar result was found in transgenic tomato fruit with over-expressed *SAMDC*, in which the levels of Spd and Spm increased, and about two-fold in *ACS* transcripts and higher ethylene level were also found as compared with the wild type fruit [39]. It was suggested that exogenous Spd might affect ethylene homeostasis by activating the expression of *ACS* during seed germination in corn.

The germinated ability of seeds seemed to be linked to the H_2_O_2_ accumulation to a critical level [7]. And H_2_O_2_ content was related with endosperm cap weakening and embryo elongation during lettuce seed germination [65]. Our data showed that exogenous Spd increased obviously the H_2_O_2_ in the plumule and radical in the process of seed imbibition compared with control; while CHA decreased the H_2_O_2_ production. This result might be closely related with the increased PAO activity in Spd-treated seed embryo and with the significant decreased PAO activity in CHA-treated one. PAO oxidized the carbon at the endo side of the N^4^-nitrogen of Spm and Spd, producing respectively N-(3-aminopropyl)-4-aminobutanal and 4-aminobutanal, as well as 1,3-diaminopropane and H_2_O_2_ [18]. Accordingly, the increased activity of PAO induced by Spd application might be a critical reason for the accumulation of H_2_O_2_ in the embryos. However, over accumulation of H_2_O_2_ easily led to oxidative damage of membrane lipids, proteins and nucleic acids [19]. The MDA content as an index of oxidative damage degree was consequently analyzed and no difference in MDA content was detected between Spd treatment and control. It was suggested that the H_2_O_2_ accumulation induced by Spd did not cause an oxidative damage to seed embryo. On the contrary, CHA significantly increased the level of MDA, which indicated that Spd deficiency resulted from exogenous CHA application caused a harmful influence on the cell membrane integrity and a stress for sprouts growth. Therefore, Spd might play an important role in cell membrane integrity maintaining. In addition, Linkies et al. [38] found that ethylene had the function on seed germination promotion by weakening endosperm cap. Therefore, it was proposed that both ethylene and H_2_O_2_ were involved in the improvement of Spd on seed germination possibly through enhancing endosperm cap weakening and stimulating the plumule and radical elongation.

## Conclusion

**Fig. 8:**
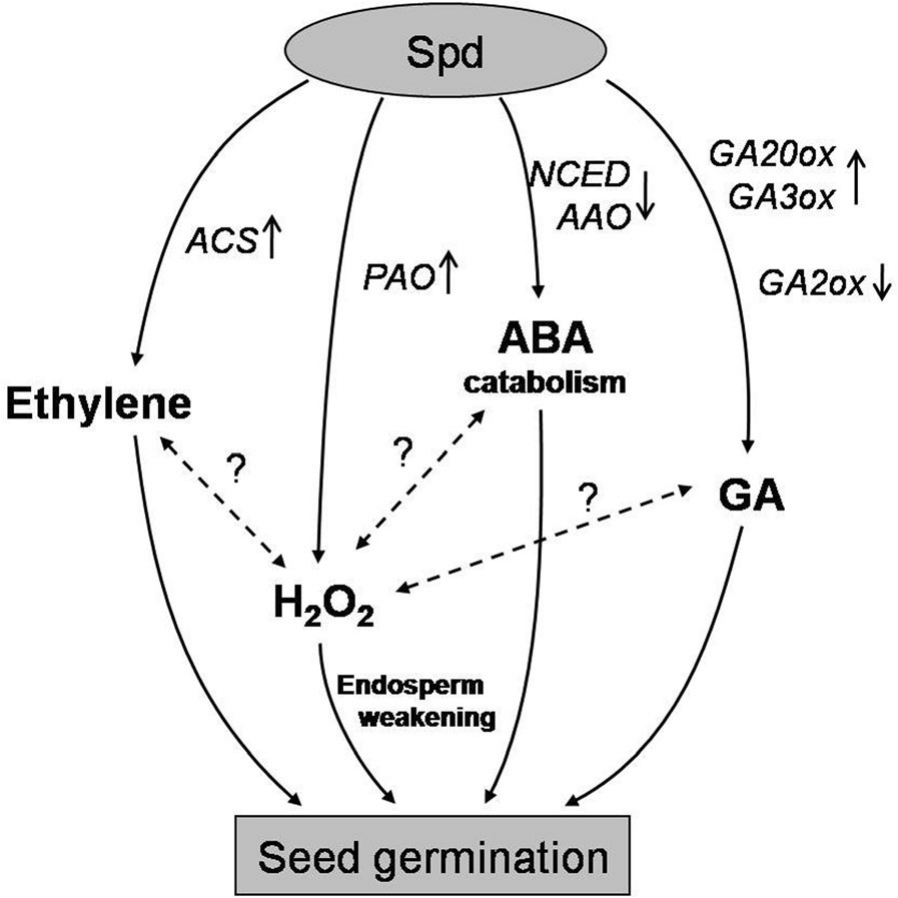
Proposed schema for the role of spermidine (Spd) in coordinating the seed germination of sweet corn. Spd increased gibberellins (GA) contents in sweet corn seeds might through improving GA biosynthetic genes expressions and depressing GA catabolic gene expression respectively. Spd decreased abscisic acid (ABA) contents in sweet corn seeds might through the depression of ABA-synthetic genes expressions. Spd also affected ethylene homeostasis by activating the expression of *ZmACS.* In addition, an increase of H_2_O_2_ content in Spd-treated seeds was found, which associated with the endosperm cap weakening and embryo elongation growth. H_2_O_2_ could also induce a decrease in ABA and an increase in ethylene directly or indirectly to accelerate the germination process [8]

That Spd contributes to fast seed germination and high seed vigor of sweet corn might be closely related with the metabolism of hormones including gibberellins, ABA and ethylene, and with the increase of H_2_O_2_ in the radical produced from Spd by PAO (Fig. 8). However, the relationship between H_2_O_2_ and hormones (gibberellins, ABA and ethylene) in the seed germination of sweet corn still needs further research.

## Additional files

Additional file 1: Effects of soaking treatments on sweet corn seedling characteristics. (PDF 92 kb)
Additional file 2: Effects of soaking treatments on hormones metabolic-related genes expressions in seed embryos. (PDF 84 kb)
Additional file 3: Main raw data presented in the manuscript. (XLSX 34 kb)

## Abbreviations

AAO: Abscisicaldehyde oxidase
ABA: Abscisic acid
ACC: 1-Amicocyclopropane-1-carboxillic-acid
ACO: ACC oxidase
ACS: ACC synthase
ADC: Decarboxylase
CHA: Cyclohexylamine
DAB: 3,3-diaminobenzidine
DAO: Diamine oxidase
DCFH_2_-DA: Dichlorodihydrofluorescein diacetate
DTT: Dithiothreitol
EDTA: Ethylenediaminetetraacetic acid
ET: Ethylene
GA: Gibberellins
GA20ox: Gibberellin 20-oxidase
GA2ox: Gibberellin 2-oxidase
GA3ox: Gibberellin 3-oxidase
GE: Germination energy
GI: Germination index
GP: Germination percentage
H_2_O_2_: Hydrogen peroxide
MDA: Malondialdehyde
NCED: 9-cis-epoxycarotenoid dioxygenase
ODC: Ornithine decarboxylase
PAO: Polyamine oxidase
PAs: Polyamines
PLP: Pyridoxal phosphate
PMSF: Phenylmethanesulfonyl fluoride
Put: Putrescine
SAMDC: S-adenosylmethionine decarboxylase
Spd: Spermidine
SPDS: Spermidine synthase
Spm: Spermine
SPMS: Spermine synthase
TBA: Thiobarbituric acid
TCA: Trichloroacetic acid
VI: Seed vigor index
ZEP: Zeaxanthin epoxidase
