# Supplementary material for: Exogenous spermidine improves seed germination of sweet corn via involvement in phytohormone interactions, H2O2 and relevant gene expression
Source: BMC Plant Biol. 2017 Jan 3;17:1. doi: 10.1186/s12870-016-0951-9 (PMC5209872; doi:10.1186/s12870-016-0951-9)
Supplement: Additional file 1: — Effects of soaking treatments on sweet corn seedling characteristics. (PDF 92 kb) [file 12870_2016_951_MOESM1_ESM.pdf]

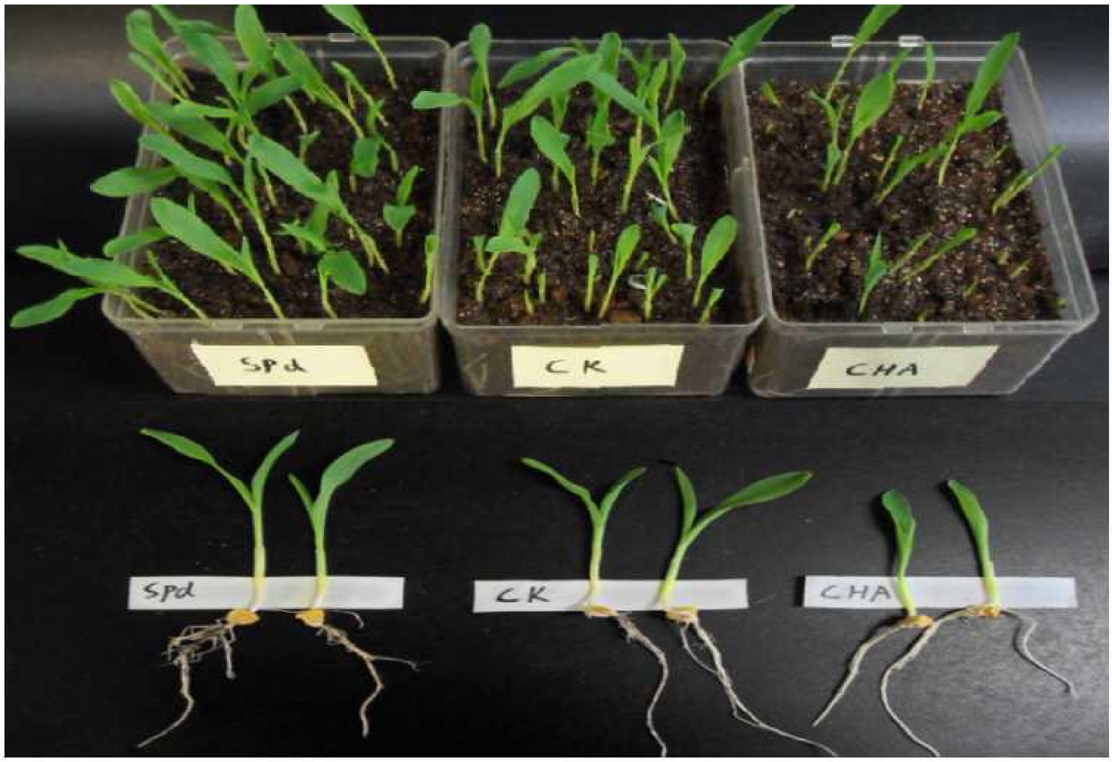

Spd **Control** **CHA**

Treatment

Additional file 1: Effects of presoaking treatments on sweet corn seedling characteristics. Control, seeds presoaking with distilled water; Spd, seeds presoaking with spermidine; CHA, seeds presoaking with cyclohexylamine. Seeds were imbibed in peat substrate moistened with water at 25°C for 7 days.
