# Supplementary material for: Exogenous spermidine improves seed germination of sweet corn via involvement in phytohormone interactions, H2O2 and relevant gene expression
Source: BMC Plant Biol. 2017 Jan 3;17:1. doi: 10.1186/s12870-016-0951-9 (PMC5209872; doi:10.1186/s12870-016-0951-9)
Supplement: Additional file 2: — Effects of soaking treatments on hormones metabolic-related genes expressions in seed embryos. (PDF 84 kb) [file 12870_2016_951_MOESM2_ESM.pdf]

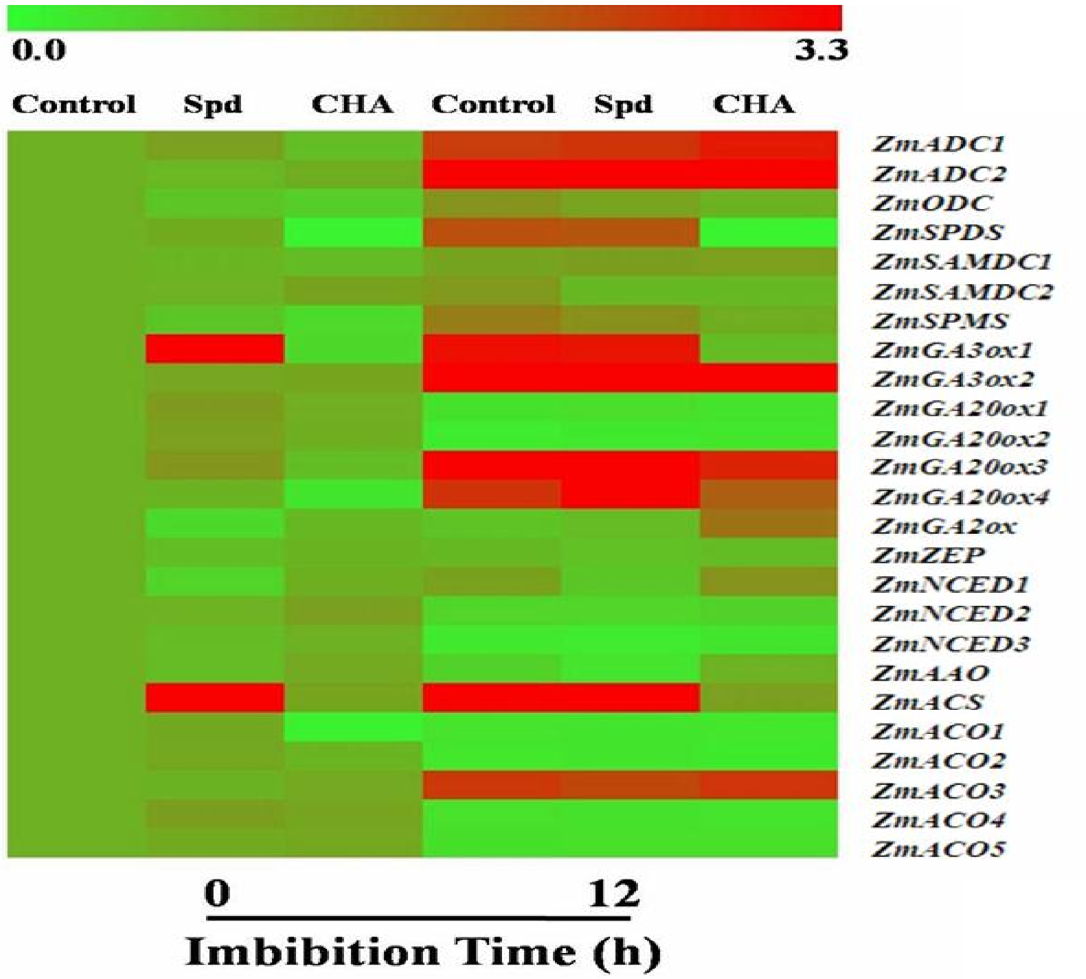

Additional file 2: Effects of presoaking treatments on hormones metabolic-related genes expression in sweet corn seed embryos. Control: presoaking with distilled water, Spd: presoaking with spermidine, CHA: presoaking with cyclohexylamine. The seed embryos were isolated from seeds after 0 or 12 h of imbibition time in rolled towels moistened with water at 25°C. ADC: Arginine decarboxylase; ODC: Ornithine decarboxylase; SPDS: Spermidine synthase ; SAMDC : S-adenosylmethionine decarboxylase; SPMS: Spermine synthase; GA3ox: Gibberellin 3-oxidase; GA20ox: Gibberellin 20-oxidase; GA2ox: gibberellin 2-oxidase; ZEP: Zeaxanthin epoxidase; NCED: 9-cis-epoxycarotenoid dioxygenase; AAO: Abscisicaldehyde oxidase; ACS: 1-Amicocyclopropane-1-carboxillic-acid (ACC) synthase; ACO: ACC oxidase. Relative expression values were normalized respect to the sample of control at 0 hour of imbibition time. Results are representative of three independent experiments. Error bars denote SE (n = 3) of biological replicates within an experiment.
